# The histone acetyltransferase HBO1 functions as a novel oncogenic gene in osteosarcoma

**DOI:** 10.7150/thno.55655

**Published:** 2021-03-04

**Authors:** Yan-yang Gao, Zhuo-yan Ling, Yun-Rong Zhu, Ce Shi, Yin Wang, Xiang-yang Zhang, Zhi-qing Zhang, Qin Jiang, Min-Bin Chen, Shuofei Yang, Cong Cao

**Affiliations:** 1 Department of Pediatrics, Xinyi people's Hospital, Xinyi, China.; 2 Department of Orthopedics, the Second Affiliated Hospital of Soochow University, Suzhou, China.; 3 Department of Orthopedics, The Affiliated Jiangyin Hospital of Medical College of Southeast University, Jiangyin, China.; 4 Department of Orthopedics, The Affiliated Suqian Hospital of Xuzhou Medical University, Suqian, China.; 5 Jiangsu Key Laboratory of Neuropsychiatric Diseases and Institute of Neuroscience, Soochow University, Suzhou, China.; 6 The Fourth School of Clinical Medicine, Nanjing Medical University, Nanjing, China.; 7 Department of Radiotherapy and Oncology, Affiliated Kunshan Hospital of Jiangsu University, Kunshan, China.; 8 Department of Vascular Surgery, Renji Hospital, School of Medicine, Shanghai Jiaotong University, Shanghai, China.; 9 North District, The Affiliated Suzhou Hospital of Nanjing Medical University, Suzhou Municipal Hospital, Suzhou, China.

**Keywords:** osteosarcoma, HBO1, histone acetylation, WM-3835

## Abstract

HBO1 (KAT7 or MYST2) is a histone acetyltransferase that acetylates H3 and H4 histones.

**Methods:** HBO1 expression was tested in human OS tissues and cells. Genetic strategies, including shRNA, CRISPR/Cas9 and overexpression constructs, were applied to exogenously alter HBO1 expression in OS cells. The HBO1 inhibitor WM-3835 was utilized to block HBO1 activation.

**Results:** *HBO1* mRNA and protein expression is significantly elevated in OS tissues and cells. In established (MG63/U2OS lines) and primary human OS cells, shRNA-mediated HBO1 silencing and CRISPR/Cas9-induced HBO1 knockout were able to potently inhibit cell viability, growth, proliferation, as well as cell migration and invasion. Significant increase of apoptosis was detected in HBO1-silenced/knockout OS cells. Conversely, ectopic HBO1 overexpression promoted OS cell proliferation and migration. We identified ZNF384 (zinc finger protein 384) as a potential transcription factor of HBO1. Increased binding between ZNF384 and HBO1 promoter was detected in OS cell and tissues, whereas ZNF384 silencing via shRNA downregulated HBO1 and produced significant anti-OS cell activity. *In vivo*, intratumoral injection of HBO1 shRNA lentivirus silenced HBO1 and inhibited OS xenograft growth in mice. Furthermore, growth of HBO1-knockout OS xenografts was significantly slower than the control xenografts. WM-3835, a novel and high-specific small molecule HBO1 inhibitor, was able to potently suppressed OS cell proliferation and migration, and led to apoptosis activation. Furthermore, intraperitoneal injection of a single dose of WM-3835 potently inhibited OS xenograft growth in SCID mice.

**Conclusion:** HBO1 overexpression promotes OS cell growth *in vitro* and *in vivo.*

## Introduction

Osteosarcoma (OS) is an aggressive malignant bone tumor arising from the primitive transformed cells with mesenchymal origin 1, 2. It is the most common histological form of primary bone cancer, and it is mainly detected in teenagers and young adults 3. It comprises 2.4% of all malignancies in pediatric patients, and about 20% of all primary bone tumors 3-5. The long-term overall survival rate of OS has improved significantly since the late 20th century (65-70%) 6, and the current clinical OS treatments include combined chemotherapy, radiation, and tumor resection 7, 8. For the patients with advanced OS, including those with metastatic or recurrence OS, the prognosis is far from satisfactory 6. As a result, novel molecular pathogenesis and targeted therapeutics for OS have been the focuses of recent studies 9-14.

The epigenetic mechanism has been suggested to play a role in the cause of OS pathogenesis and progression 15. Among the epigenetic alternations, histone modification through reversible acetylation is crucial for gene expression regulation 16-18. Two enzymes, histone acetyltransferase (HAT) and histone deacetylase (HDAC), are responsible for histone acetylation 18, 19. Dysregulation of histone acetylation is associated with OS tumorigenesis and progression 15, 20, 21.

HBO1 (KAT7 or MYST2) is a HAT that acetylates H4 and H3 histones at different lysine residues 22. It consists of an N-terminal domain and a C-terminal MYST domain, which are required for acetyl-CoA binding and acetylation 23. HBO1 is critical in regulating a number of key cellular behaviors and physiological functions, including gene transcription, protein ubiquitination, immune regulation, stem cell pluripotent, self-renewal maintenance, and embryonic development 23-28, and it also forms complexes with native subunits and cofactors 22, 23. Complexes formed by HBO1 and BRPF scaffold (BRPF1, BRD1/BRPF2 or BRPF3) are capable of directing HBO1 specificity toward histone H3K14 acetylation (H3K14ac). Moreover, HBO1 complexes containing JADE (JADE1, JADE2 and JADE3) can also direct HBO1 specificity toward histone H4 acetylation 24, 25, 29. H3K14ac by HBO1 facilitates the activation of replication origins, and histone H4 acetylation facilitates chromatin loading of minichromosome maintenance protein complex (MCM) to promote DNA replication licensing 24-28.

HBO1 binds directly to cdt1 (Chromatin licensing and DNA replication Factor 1), a protein required for G1 phase cell cycle progression 22, 30, to promote histone acetylation in cell cycle progression and cell proliferation. Additionally, HBO1 acts as a positive regulator of centromeric CENPA (Centromere Protein A) by preventing SUV39H1-mediated centromere inactivation 31. Niida *et al.,* found that ATR-dependent HBO1 phosphorylation induced by ultraviolet irradiation promoted its localization to DNA damage sites, which is essential for XPC recruitment and nucleotide excision repair 32.

Recent studies have proposed that HBO1 plays an oncogenic role in human cancers 22, 23, 33. Wang *et al.,* demonstrated that HBO1 overexpression in gastric cancer is negatively correlated with patients' survival 34. In bladder cancer cells, HBO1 is involved in Wnt/beta-catenin signaling and cancer cell proliferation 35. Another study by MacPherson *et al.* has shown that HBO1 HAT domain is essential for the acetylation of H3K14 (H3K14ac). The latter promoted the processivity of RNA polymerase II to maintain high expression of key genes (*MYLK*,* HOXA9* and others) in leukemia stem cells 33. While genetic silencing or pharmacological inhibition of HBO1 potently inhibited leukemia stem cell progression 33. Kueh and colleagues, however, reported that HBO1 does not have an essential role in cell proliferation and DNA replication in HEK293T, MCF7, or HeLa 36. In this study, we tested the expression and potential function of HBO1 in human OS 36, and we show that overexpressed HBO1 acts as a novel oncogenic gene essential for OS cell tumorigenesis and progression.

## Methods

### Chemicals and reagents

Cell Counting Kit-8 (CCK-8) was provided by Dojindo Co. (Kumamoto, Japan). Puromycin and polybrene were provided by Sigma-Aldrich Chemicals (St. Louis, Mo). Antibodies for HBO1 (#58418), cleaved caspase-3 (#9664), acetyl-Histone H3 at Lys14 (H3K14ac, #7627), Histone H3 (#4499), acetyl-Histone H4 at Lys12 (H4K12ac, #13944), Histone H4 (#2935), acetyl-Histone H4 at Lys5 (H4K5ac, #8674), cleaved-poly (ADP-ribose) polymerase (PARP) (#5625), cleaved-caspase-9 (#20750), Caspase-3 (#9668), Caspase-9 (#9508), PARP (#9532) and Tubulin (#2125) were obtained from Cell Signaling Tech China (Shanghai, China). A Zinc finger protein 384 (ZNF 384) antibody was provided by Abcam (ab176689, Shanghai, China). Caspase inhibitors, z-DEVD-fmk and z-VAD-fmk, were provided by Sigma-Aldrich Chemicals. The HBO1 inhibitor WM-3835, N'-(4-fluoro-5-methyl-[1,1'-biphenyl]-3-carbonyl)-3-hydroxybenzenesulfonohydrazide was synthesized by Min-de Biotech (Suzhou, China) based on the described protocol 33. RNA reagents, Lipofectamine 2000, and other transfection reagents were provided by Thermo-Fisher Invitrogen (Shanghai, China). All primers, sequences, constructs, and vectors were designed and provided by Genechem Co. (Shanghai, China), unless mentioned otherwise.

### Cell culture

Primary human OS cells from Dr. Ji at Nanjing Medical University 37, 38 were derived from two written-informed consent OS patients. Patients received no chemotherapy and radiotherapy before surgery. OS tumor tissues were washed, minced, and incubated in collagenase I solution (Gibco, Boston, MA). Cell pellets were isolated and cultured in described medium 39. Primary OS cells were named as pOS-1 and pOS-2. The established human OS cell lines, U2OS and MG-63, were purchased from Cell Bank of Shanghai Institute of Biological Science (Shanghai, China). The primary human osteoblasts were provided by Dr. Ji as well 40, 41. Human osteoblasts were differentiated and cultured as described 42, 43. The protocols of using primary human cells were approved by IACUC and Ethics committee of Nanjing Medical University.

### Human tissues

OS tissues and the surrounding normal bone tissues from a set of ten (10) written-informed primary OS patients were provided by authors' institutions. These patients received no chemotherapy and radiotherapy before surgery. Tissues were incubated with the described lysis buffer 37 and stored in liquid nitrogen. The written-informed consent was obtained from each participant. The protocols of this study were approved by the Ethic Committee of Soochow University according to Declaration of Helsinki.

### Western blotting

The detailed protocols of Western blotting analyses were described in details in our previous studies 44-46. When testing different proteins, the exact same set of lysates were run in parallel “sister” gels.

### Quantitative real time-PCR (qPCR)

Total RNA was extracted by TRIzol reagents and was reversely transcripted to cDNA 47. qPCR was performed by an ABI Prism 7500 system using a SYBR GREEN PCR Master Mix (Applied Biosystems, Shanghai, China). The product melting temperature was always calculated. *Glyceraldehyde-3-phosphatedehydrogenase* (*GAPDH*) was tested as the internal control and reference gene. The quantification of targeted mRNA was done by 2^-∆∆*C*t^ method. The mRNA primers were listed in **Table-S1**. Other mRNA primers were verified and provided by Genechem (Shanghai, China).

### shRNA-mediating gene silencing

Two shRNAs targeting non-overlapping sequences of human HBO1, shHBO1-a and shHBO1-b (sequences listed in **Table-S1**), were designed and verified by Genechem Co. The shRNAs were sub-cloned into GV248 (hU6-MCS-Ubiquitin-IRES-puromycin) vector (Genechem), and the two constructs were individually transfected to HEK-293T cells together with the lentivirus package plasmid mix (Genechem). OS cells were seeded into six-well plates (0.8×10^5^ cells per well) in polybrene-containing complete medium. HBO1 shRNA lentivirus was added to cultured OS cells. To select stable cells, puromycin (5.0 µg/mL) was added in complete medium, and cells were cultured for five passages (10-12 days). HBO1 silencing in stable cells was assessed by qPCR and Western blotting assays. Control cells were transfected with lentivirus encoding scramble control shRNA (“shC”). ZNF384 shRNAs (sequences listed in **Table-S1**) and stable cell selection were carried out similarly.

### HBO1 knockout

The small guide RNA (sgRNA) targeting human *HBO1* (Target DNA sequence: GATGAACGAGTCTGCCGAAG, PAM sequence PAM Sequence: AGG) was inserted into the lenti-CRISPR-GFP-puro plasmid 48. OS cells were seeded into six-well plates (0.8×10^5^ cells per well) in polybrene-containing complete medium. The construct was transfected to OS cells by Lipofectamine 2000 (Thermo-Fisher Invitrogen, Shanghai, China). GFP-positive OS cells were sorted by FACS, and then distributed into 192-well plates to achieve single cells. Cells were further subjected to HBO1 knockout (KO) screening. HBO1 KO stable cells were achieved via the selection by puromycin (5 µg/mL) and then verified by qPCR and Western blotting analyses.

### Forced HBO1 overexpression

The full-length *HBO1 cDNA* sequence was synthesized by Genechem Co. (Shanghai, China), and it was sub-cloned into a GV369 gene expression lentiviral vector (Genechem). The construct and the lentivirus package plasmid mix were co-transfected into HEK-293T cells to generate HBO1-expression lentivirus. OS cells were seeded into six-well plates (0.8×10^5^ cells per well) in polybrene-containing complete medium. The viruses were filtered, enriched, and added to cultured OS cells. To select stable cells, puromycin was added in the culture medium. HBO1 overexpression in the stable cells was then verified by qPCR and Western blotting analyses.

### Cell viability and death assays

Briefly, cells with applied genetic modifications or treatments were seeded into 96-well plates at 3×10^3^ cells per well. After 96 h incubation, in each well 10 µL CCK-8 reagent was added for 3 h. Later, CCK-8's optical density (OD) value was recorded at 450 nm. Cell death was tested by Trypan blue staining using an automatic cell counter.

### Cell migration and invasion

OS cells with the applied genetic modifications or treatments were seeded at 5×10^4^ cells per chamber (in serum-free medium) into the upper surfaces of “Transwell” chambers (BD Biosciences, Shanghai, China). The complete medium with 12% FBS was added into the lower compartments of the chambers. Following 24 h incubation, OS cells that migrated through the membrane were fixed by using 4% paraformaldehyde and co-stained with crystal violet. Cells were then observed and counted under a microscope (magnification: ×100). For cell invasion assays, the chamber surface was coated with Matrigel (Sigma).

### EdU staining

OS cells were seeded into six-well plates at 1.2×10^5^ cells per well and were cultured for 48 h. An EdU (5-ethynyl-20-deoxyuridine) Apollo-567 Kit (RiboBio, Guangzhou, China) was applied to quantify cell proliferation according to the attached protocols. EdU and DAPI fluorescent dyes were added to OS cells and the cells were visualized under a fluorescent microscope (Leica, Shanghai, China). Five random views (n = 5) with a total of 1 000 cells per each condition were used to calculate the average EdU ratio (% vs. DAPI).

### TUNEL staining

OS cells with the applied genetic modifications or treatments were seeded into six-well plates at 1.2×10^5^ cells per well and cultured for 72 h. Cells were then stained with TUNEL (Invitrogen) for 4h under the dark. Nuclei were co-stained with DAPI and visualized under a fluorescent microscope. Five random views (n = 5) contained a total of 1, 000 cells from each condition were used to calculate the average TUNEL ratio (% vs. DAPI).

### Single stranded DNA (ssDNA) ELISA

In apoptotic cells, DNA breaks would lead to the accumulation of ssDNA. Briefly, 48 h after applied treatment, 30 µg of cell lysates per treatment were analyzed by ssDNA ELISA kit (Roche Diagnostics, Shanghai, China) based on the attached protocol. The ssDNA ELISA absorbance in each well was tested at 450 nm.

### FACS

OS cells with applied genetic modifications were seeded into six-well plates at a density of 1.2×10^5^ cells per well. After applied treatments, cells were harvested, washed, and resuspended in the binding buffer with Annexin V-FITC and propidium iodide (PI) (both at 5 µg/mL). Cells were then analyzed by a flow cytometer (Becton Dickinson, Shanghai, China) using BD FACSDivaTM software. Annexin V-positive cells were gated as apoptotic cells, and the ratio was recorded. To test cell cycle progression, cells were stained only with PI.

### Caspase-3 activity

OS cells with the applied genetic modifications or treatments were seeded into six-well plates at a density of 1.2 × 10^5^ cells per well. Cells were collected and lysed with cell lysis buffer. Caspase-3 activation was measured fluorometrically through a caspase-3 fluorescent assay kit (Sigma-Aldrich) with active caspase-3 substrate peptides Ac-DEVD-AMC (7-amido-4-methyl coumarin) fluorogenic substrate. It was tested under a spectrofluorometer at an excitation wavelength of 380 nm and an emission wavelength of 450 nm.

### Mitochondrial depolarization

With mitochondrial membrane potential (MMP) reduction and mitochondrial depolarization, the fluorescent probe JC-1 aggregates are decomposed into a monomer form, and the emitted fluorescence changes from red to green. Cells with applied genetic modifications or treatments were seeded into six-well plates at a density of 1.2×10^5^ cells per well and incubated with JC-1 at room temperature for 20 min under the dark. The JC-1 green monomers intensity (488 nm) was tested with a flow cytometer (Becton Dickinson). The representative JC-1 images integrating both green and red fluorescence channels were presented.

### Chromatin immunoprecipitation (ChIP) assay

As reported 49, cells or fresh human tissues were first cross-linked with 1% formaldehyde, and glycine solution is added for quenching. Afterwards, cells or tissues were washed and lysed using the lysis buffer. Lysate samples were treated with a MisonixSonicator 3000 Homogenizer 49 to fragment genomic DNA (200-800 bp). After centrifugation, the supernatant was removed and ChIP Dilution Buffer was added. Lysates were then pre-cleared, and an anti-ZNF384 antibody was added. Detailed protocols for ChIP were reported previously 49. At last, ZNF384-bound DNA was eluted from the protein A/G agarose with elution buffer, and NaCl was added to eliminate cross-linking between proteins and genomic DNA. DNA containing HBO1 promoter was analyzed via quantitative PCR with the primers at the promoter region of HBO1 (Genechem, Shanghai, China). For all ChIP assay experiments, negative control regions were tested to exclude the possible non-specific binding.

### Small interfering RNA (siRNA)

The siRNAs targeting SOX10, PITX3, ZNF384, and SP2 were designed, synthesized, and verified by Genechem (Shanghai, China). The sequences were listed in **Table-S1**. OS cells were seeded into six-well tissue culture plate at 0.8×10^5^ cells per well (50-60% confluence). Cells were transfected with 200 nM of targeted siRNA or the scramble control siRNA (“siR-C”) by Lipofectamine 3000 (Thermo-Fisher Invitrogen, Shanghai, China) for 48 h. Over 80% knockdown efficiency of targeted mRNA was achieved (tested by qPCR assays).

### Xenograft assay

The severe combined immunodeficient (SCID) mice (18-19g, female) were obtained from Experimental Animal Center of Soochow University (Suzhou, China). pOS1 cells (5×10^6^ cells per mouse, in Matrigel-containing serum free medium) were subcutaneously (*s.c.*) injected to the right flanks. Tumor-bearing SICD mice were randomly assigned into different groups (7-10 mice per group). Tumor volumes and the mice body weights were recorded every seven days. The animal studies were approved by Institutional Animal Care and Use Committee (IACUC) and Ethics Committee of Soochow University.

### Statistical analyses

*In vitro* experiments were repeated at least three times, and similar results were obtained. The quantitative data of normal distribution were presented as mean ± standard deviation (S.D.). Statistical analyses were carried out by one-way ANOVA with the Scheffe and Tukey Test (SPSS 23.0, SPSS Co. Chicago, IL). When comparing the significance between two treatment groups, a two-tailed unpaired T test was utilized (Excel 2007, Microsoft Co. Seattle, Washington). *P*-values < 0.05 were considered as statistically significant differences.

## Results

### HBO1 upregulation in human OS

**Figure 1:**
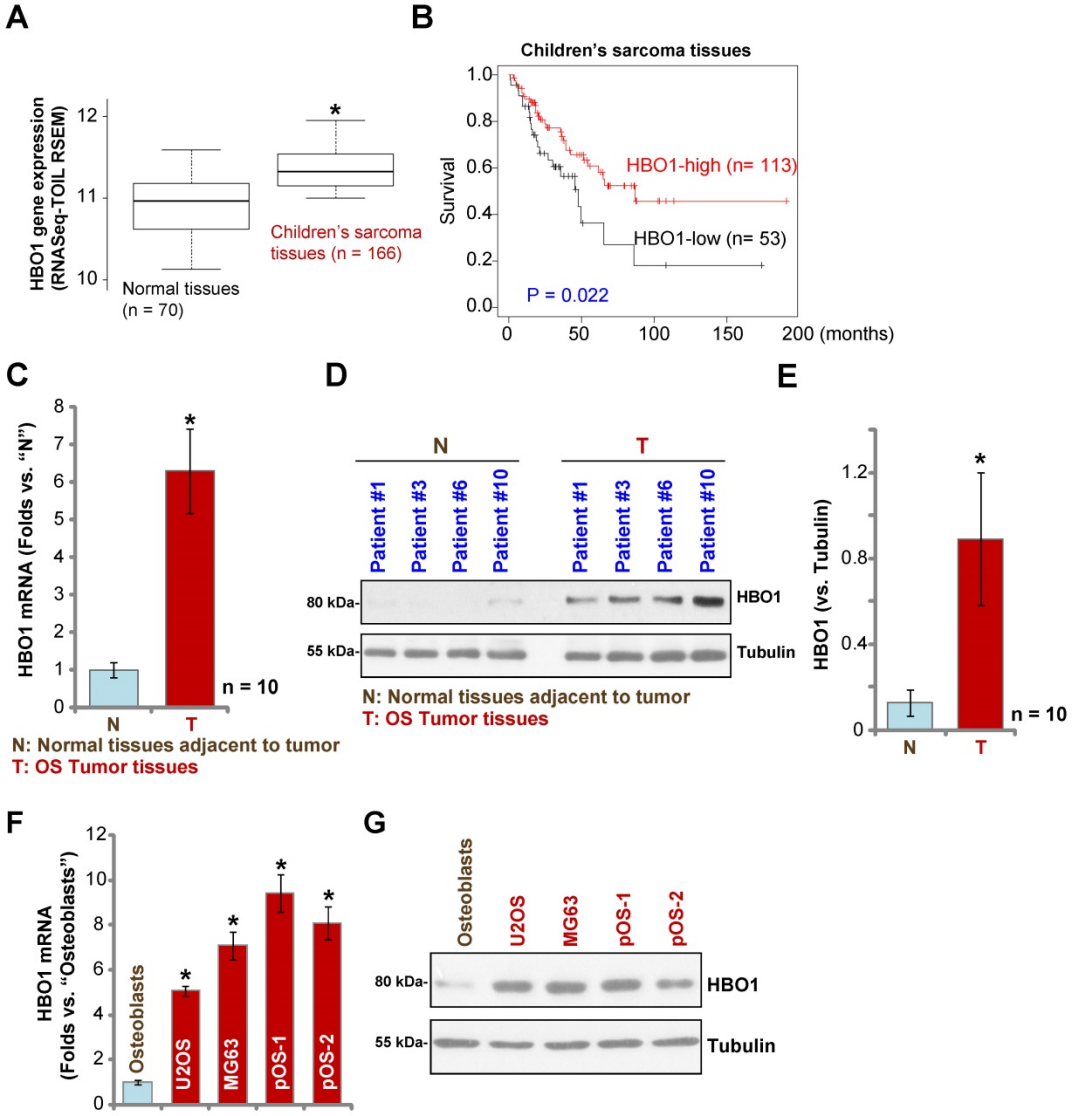
** HBO1 upregulation in human OS. The TARGET Pan-Cancer (PANCAN) database shows *HBO1* expression (RNASeq-TOIL RSEM) in 166 cases of children's sarcoma tissues and 70 cases of normal adjacent tissues** (**A**). Kaplan Meier Survival analyses of HBO1-low (n = 53) and HBO1-high (n = 113) children's sarcoma patients (**B**). *HBO1* mRNA and protein expression in OS tumor tissues (“T”) and in normal tissues adjacent to tumor (“N”) of ten (n = 10) primary human OS patients was shown (**C-E**). *HBO1* mRNA and protein expression in established OS cell lines (U2OS and MG63), primary human OS cells (pOS-1 and pOS-2), as well as in primary human osteoblasts (“Osteoblasts”) were shown (**F and G**). Data were presented as mean ± standard deviation (SD). * *P* < 0.05 vs. “N” tissues/Osteoblasts (C-G).

Over 60% of the common histological subtypes of bone sarcoma are OS 3. We first searched the TARGET Pan-Cancer (PANCAN) database and examined *HBO1* expression in children's sarcoma tissues using the UCSC Xena project, and then we screened RNASeq data of *HBO1* in sarcoma tissues. As demonstrated, *HBO1* mRNA expression in children's sarcoma tissues (n = 166) is significantly upregulated [*P* < 0.05 vs. surrounding normal tissues (n = 70)] (**Figure 1A**). Importantly, high expression of *HBO1* mRNA in human sarcoma tissues is associated with low overall survival (**Figure 1B**).

To confirm the bioinformatics results, we tested HBO1 expression in human OS tissues. Tissue specimens from a set of ten OS patients were obtained. To test mRNA expression, qPCR was performed. As shown in **Figure 1C**, *HBO1* mRNA expression in OS tumor tissues (“T”) was over six-fold than that in normal tissues adjacent to tumor (“N”) (*P* < 0.05 vs. “N” tissues). After testing HBO1 protein expression by Western blotting analyses, we confirmed the dramatic HBO1 protein upregulation in OS tumor tissues from four representative patients (Patient #1/#3/#6/#10) (**Figure 1D**). When combining all human tissues' blotting data, we found that HBO1 protein level in OS tumor tissues is significantly upregulated (*P* < 0.05 vs. “N” tissues) (**Figure 1E**).

HBO1 expression in OS cells was tested as well. In established OS cell lines, U2OS and MG-63, as well as in the primary human OS cells, pOS-1, and pOS-2 (derived from two OS patients), *HBO1* mRNA expression was significantly higher than that in the primary human osteoblasts (“Osteoblasts” 40, 41) (*P* < 0.05, **Figure 1F**). Furthermore, HBO1 protein upregulation was detected in established OS cells and primary human OS cells (**Figure 1G**) when compared to the low expression in primary human osteoblasts (**Figure 1G**). These results demonstrated that HBO1 is upregulated in OS.

### HBO1 shRNA inhibits OS cell progression *in vitro*

**Figure 2:**
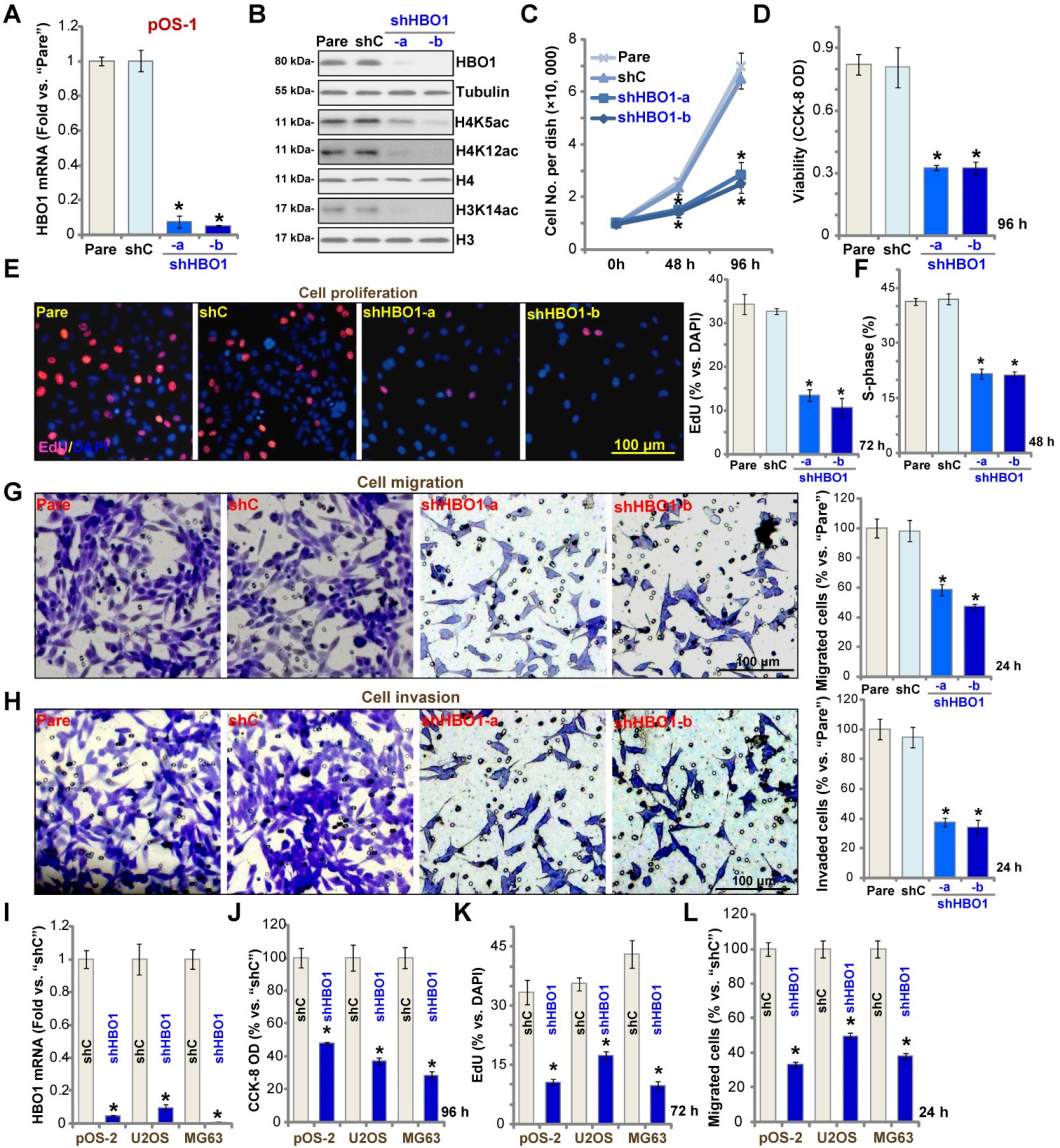
** HBO1 silencing inhibits OS cell progression *in vitro*.** Stable primary OS cells (pOS-1 and pOS-2) and established OS cell lines (U2OS and MG63) that expressed the HBO1 shRNA (shHBO1-a/shHBO1-b) or the scramble control shRNA (“shC”) were established. After cultured for applied time periods, expression of *HBO1* mRNA and listed proteins was shown (**A**, **B**, and **I**); Cell growth (**C**), viability (**D** and **J**), proliferation (**E** and **K**), S-phase cell percentage (**F**), cell migration (**G** and **L**) and invasion (**H**) were tested by the listed assays, with results quantified. For nuclear EdU staining, five random views (n = 5) of total 1, 000 cell nuclei per each condition were used to calculate the average EdU ratio (% vs. DAPI, same for all EdU studies). For “Transwell” and “Matrigel Transwell” assays, five random microscopy views were included to calculate the average number of migrated or invaded cells in each condition (same for all “Transwell” assays). For the *in vitro* cellular functional studies, the exact same number of viable cells with the applied genetic modifications was initially seeded into each well/dish (“Day-0”/“0h”, same for all figures), and cells were cultured for indicated time periods. “Pare” stands for the parental control OS cells. The data were presented as mean ± standard deviation (SD, n = 5). * *P* < 0.05 vs. “sh-C” cells. The experiments were repeated five times with similar results obtained. Scale bar = 100 µm (**E**, **G** and **H**).

To study the potential role of HBO1 in human OS cells, shRNA strategy was applied. Two lentiviral shRNAs targeting non-overlapping sequences of human HBO1, shHBO1-a and shHBO1-b, were individually transduced to pOS-1 cells. Stable cells were established by the selection of puromycin containing medium. qPCR assay results in **Figure 2A** confirmed that *HBO1* mRNA decreased over 90% in stable pOS-1 cells containing HBO1 shRNA. HBO1 protein levels were significantly downregulated as well (**Figure 2B**), leading to dramatic inhibition of H4 acetylation (H4K5ac and H4K12ac) and H3K14ac 33 (**Figure 2B**). Expressions of HBO1-dependent mRNAs, *MYLK* and* HOXA9* were downregulated in HBO1 shRNA-expressing OS cells (**Figure S1A**). As compared to the parental control cells, the growth of HBO1 shRNA-expressing pOS-1 cells was significantly inhibited (**Figure 2C**). CCK-8 assay results in **Figure 2D** demonstrated that cell viability was robustly decreased in HBO1-silenced pOS-1 cells.

Miotto* et al.,* have shown that HBO1 directly interacts with the replication licensing factor Cdt1 50 and its histone acetylase activity is required for DNA replication licensing 51. Conversely, Cdt1 phosphorylation by JNK1 inhibited Cdt1-HBO1 association and blocked replication licensing 26. Evidenced by significantly decreased percentage of EdU-positive nuclei, pOS-1 cell proliferation was inhibited by shRNA-mediated knockdown of HBO1 (**Figure 2E**). The PI-FACS assay demonstrated that HBO1 knockdown induced reduction of S-phase cells (**Figure 2F**).

Next, “Transwell” and “Matrigel Transwell” assays were performed to test cell migration and invasion, respectively. We found that HBO1 shRNA significantly inhibited migration (**Figure 2G**) and invasion (**Figure 2H**) of pOS-1 cell. For the “Transwell” assays, cells were incubated for only 24h to exclude the possible influence of cell growth/proliferation change. Together, we reported here HBO1 silencing potently inhibited pOS-1 cell viability, growth, proliferation, as well as cycle cycle progression, cell migration and invasion. The scramble control shRNA (“shC”), as expected, did not affect HBO1 expression (**Figure 2A and B**) and pOS-1 cell functions (**Figure 2C**-**H**).

In the established human OS cell lines U2OS and MG63, and in primary OS cells-derived another patient (pOS-2), transfection of HBO1 shRNA lentivirus (shHBO1-a) led to dramatic *HBO1* mRNA (**Figure 2I**) and protein (**Figure S1B**) downregulation. Functional studies demonstrated that HBO1 shRNA largely inhibited cell viability (**Figure 2J**), proliferation (**Figure 2K**), and migration (**Figure 2L**). These results indicated that HBO1 silencing inhibits OS cell progression *in vitro*.

### HBO1 shRNA provokes OS cell apoptosis

**Figure 3:**
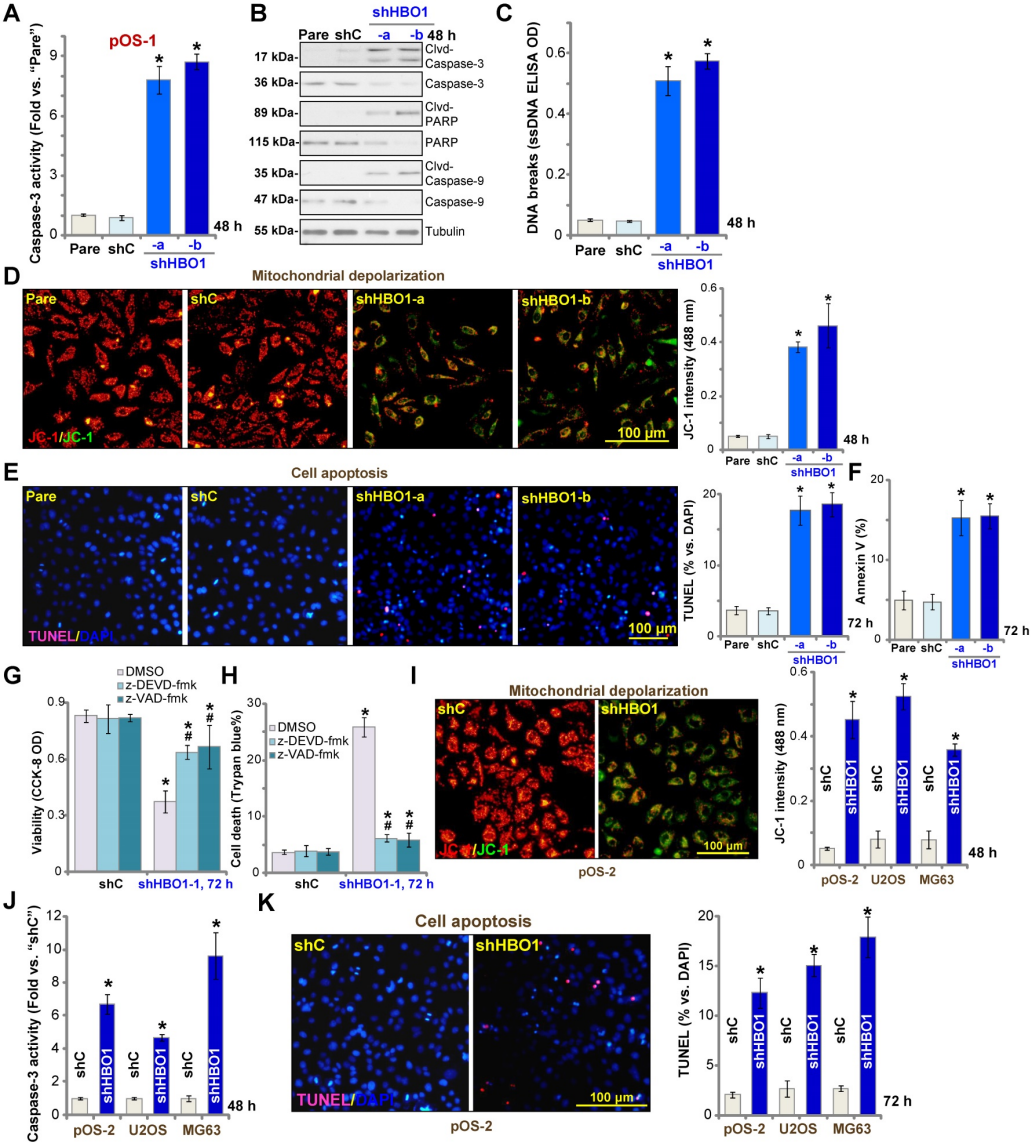
** HBO1 shRNA provokes OS cell apoptosis.** Stable primary OS cells (pOS-1 and pOS-2), as well as the established OS cell lines (U2OS and MG63) that expressed HBO1 shRNA (shHBO1-a/shHBO1-b) or the scramble control shRNA (“shC”) were established and cultured for applied time periods, caspase activation (**A**, **B** and **J**), single strand DNA (ssDNA) contents (**C**), and mitochondrial depolarization (by recording JC-1 green monomers, **D** and **I**) were tested; Cell apoptosis was tested by nuclear TUNEL staining (**E** and **K**) and Annexin V FACS (**F**) assays. Stable pOS-1 cells with shHBO1-a or shC were treated with z-DEVD-fmk (50 μM), z-VAD-fmk (50 µM) or vehicle control (0.1% of DMSO), and cultured for 96h. Cell viability and death were tested by CCK-8 (**G**) and Trypan blue staining (**H**) assays, respectively. For nuclear TUNEL staining assays, five random views of a total 1, 000 cells per each condition were included to calculate the average TUNEL ratio (% vs. DAPI, same for all TUNEL assays). “Pare” stands for the parental control OS cells. The data were presented as mean ± standard deviation (SD, n = 5). *P < 0.05 vs. “sh-C” cells. **^#^***P* < 0.05 vs.“DMSO” treatment (**G** and **H**). The experiments were repeated five times with similar results obtained. Scale bar = 100 µm (**D**, **E**, **I** and **K**).

The potential effect of HBO1 shRNA in cell apoptosis was studied. In pOS-1 primary cells, as HBO1 was silenced by shHBO1-a and shHBO1-b (see **Figure 2**), we discovered significantly enhanced activity of caspase-3 (**Figure 3A**) and increased cleavages of caspase-3, caspase-9, and PARP (poly ADP-ribose polymerase) (**Figure 3B**). Increased single stand DNA (ssDNA) contents, which indicates DNA breaks, were also detected in HBO1-silenced pOS-1 cells (**Figure 3C**). Evidenced by mitochondrial JC-1 green monomers accumulation, we demonstrated that mitochondrial depolarization in pOS-1 cells with HBO1 shRNAs (**Figure 3D**). These results suggested that mitochondrial apoptosis cascade was activated in HBO1-silenced OS cells 52, 53.

We further showed that TUNEL-positive nuclei ratio was significantly increased in pOS-1 cells with HBO1 shRNAs, suggesting the upregulated apoptosis level (**Figure 3E**). Additionally, the number of pOS-1 cells with positive Annexin V staining was largely increased following HBO1 silencing (**Figure 3F**), further confirming apoptosis activation. The scramble control shRNA (“shC”) failed to provoke significant apoptosis activation in pOS-1 cells (**Figure 3A**-**F**). In pOS-1 cells, two caspase inhibitors, the caspase-3 inhibitor z-DEVD-fmk and the pan caspase inhibitor z-VAD-fmk, largely attenuated shHBO1-a-induced viability (CCK-8 OD) reduction (**Figure 3G**) and cell death (by recording Trypan blue-positive cells, **Figure 3H**).

In pOS-2 primary cells and established OS cell lines (U2OS/MG63), HBO1 silencing by shHBO1-a (see **Figure 2 and Figure S1B**) induced mitochondrial depolarization (**Figure 3I**), which also activated caspase-3 (**Figure 3J**) and increased TUNEL-positive nuclei ratio (**Figure 3K**), indicating apoptosis activation. These results indicated that HBO1 silencing would provoke OS cell apoptosis.

### HBO1 knockout induces potent anti-OS cell activity

To exclude possible off-target effect of the applied HBO1 shRNAs, the CRISPR/Cas9 gene editing method was utilized to stably knockout (KO) HBO1. A lentiviral CRISPR/Cas9-HBO1-KO construct (see Methods) was transduced in pOS-1 primary cells. After KO screening and puromycin selection, two stable cell lines, koHBO1-1 and koHBO1-2, were established. As shown, *HBO1* mRNA (**Figure S2A**) and protein (**Figure S2B**) expressions were depleted in koHBO1pOS-1 cells. H4 and H3 de-acetylation (**Figure S2B**), as well as *MYLK-HOXA9* downregulation (**Figure S1C**), were detected in HBO1 KO cells. CRISPR/Cas9-induced HBO1 KO in pOS-1 cells potently inhibited cell viability (CCK-8 OD, **Figure S2C**) and proliferation (by recording EdU-positive nuclei ratio, **Figure S2D**). “Transwell” assay results in **Figure S2E** also demonstrated that cell migration was largely inhibited in koHBO1 cells.

Further studies showed that the caspase-3 activity was robustly increased in koHBO1-1 cells and koHBO1-2 cells (**Figure S2F**). HBO1 KO also induced cleavages of caspase-3, caspase-9, and PARP (**Figure S2G**) in pOS-1 cells, and accumulation of ssDNA that indicated DNA damage (**Figure S2H**). Additionally, the increased TUNEL-positive nuclei ratio confirmed the activation of apoptosis in koHBO1 pOS-1 cells (**Figure S2I**).

Similar results were observed in pOS-2 primary cells and established OS cell lines (U2OS/MG63). Stable introduction of the CRISPR/Cas9-HBO1-KO construct (“koHBO1”) almost depleted *HBO1* mRNA and protein (**Figure S2J and Figure S1D**). HBO1 KO largely inhibited cell viability (**Figure S2K**), proliferation (**Figure S2L**), and migration (**Figure S2M**). Therefore, CRISPR/Cas9-induced HBO1 KO produced significant anti-OS cell activity.

### Ectopic HBO1 overexpression promotes OS cell growth

**Figure 4:**
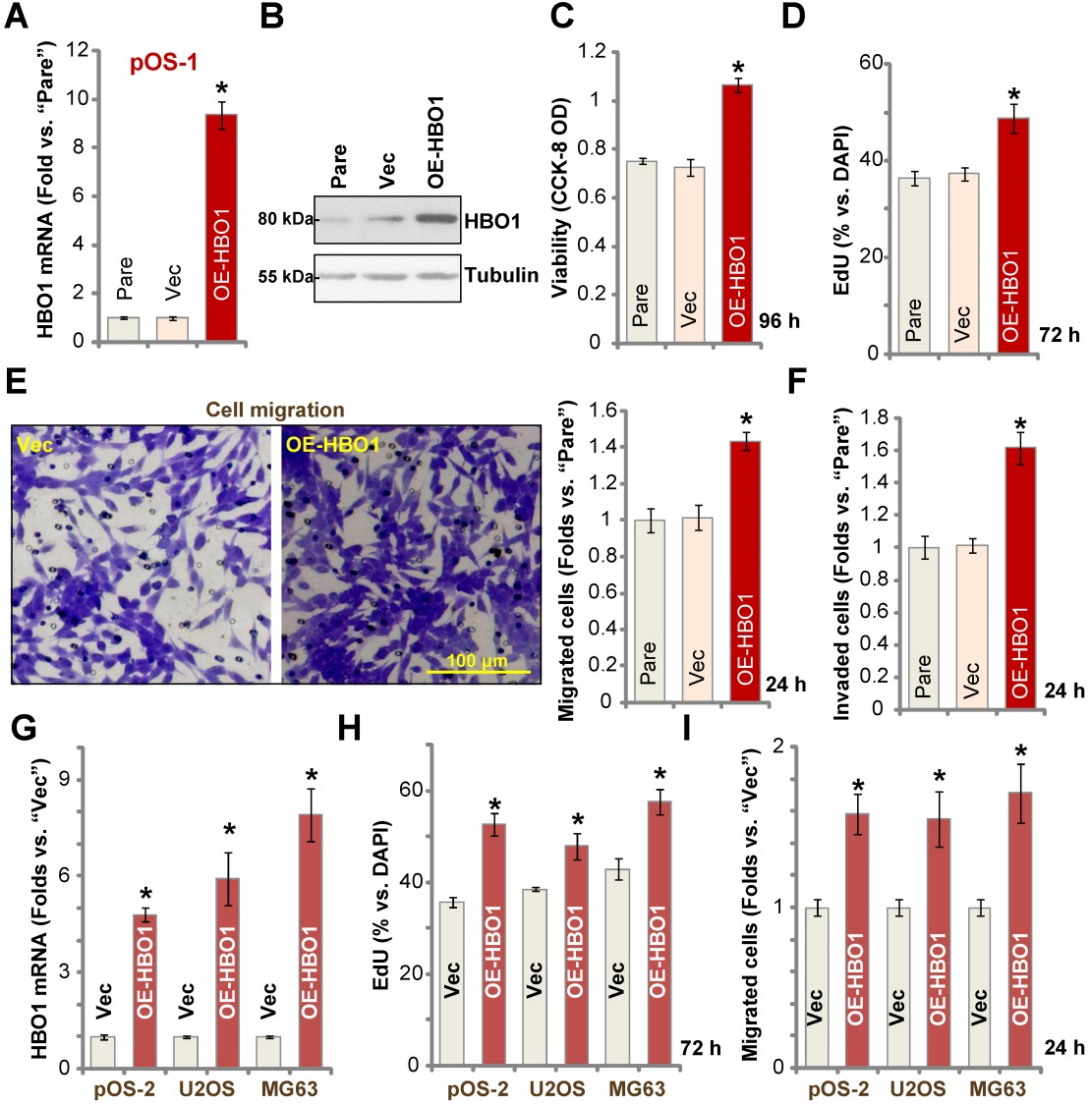
** Ectopic HBO1 overexpression promotes OS cell growth**. Stable primary OS cells (pOS-1 and pOS-2) and established OS cell lines (U2OS and MG63) with lentiviral construct encoding full-length *HBO1 cDNA* (“OE-HBO1”) or the empty vector (“Vec”), were established and cultured for applied time periods; Expression of *HBO1* mRNA and listed proteins was shown (**A**, **B** and **G**); Cell viability (CCK-8 OD, **C**), proliferation (by recording EdU-positive nuclei ratio, **D** and **H**), migration and invasion (“Transwell” assays, **E**, **F** and **I**) were tested by the listed assays, and results were quantified. “Pare” stands for the parental control OS cells. The data were presented as mean ± standard deviation (SD, n = 5). *P < 0.05 vs. “Vec” cells. The experiments were repeated five times with similar results obtained. Scale bar = 100 µm (**E**).

Because HBO1 shRNA or KO inhibited OS cell growth and induced cell apoptosis, we hypothesized that ectopic overexpression of HBO1 should exert opposite effects. Therefore, a lentiviral construct encoding full-length *HBO1 cDNA* was transduced to pOS-1 primary cells, and we established stable OE-HBO1 cells through selection. Compared to the control cells with the empty vector (“Vec”), *HBO1* mRNA levels were significantly increased (over nine folds) in OE-HBO1 cells (**Figure 4A**). Consequently, HBO1 protein expression was also upregulated (**Figure 4B**), with increased* MYLK-HOXA9* mRNA expression detected as well (**Figure S1E**). Through cell viability testing with CCK-8 assays, we confirmed that ectopic overexpression of HBO1 increased the viability of pOS-1 cells (**Figure 4C**). EdU-positive nuclei ratio was also significantly increased in the OE-HBO1cells, indicating that HBO1 overexpression promoted pOS-1 cell proliferation (**Figure 4D**, results were quantified). Ectopic overexpression of HBO1 promoted pOS-1 cell migration (**Figure 4E**) and invasion (**Figure 4F**, results were quantified). As expected, the vector control failed to affect HBO1 expression (**Figure 4A and B**) and the functions of pOS-1 cells (**Figure 4C**-**F**).

In pOS-2 primary cells and established OS cell lines (U2OS/MG63), stable transfection of the HBO1 construct increased *HBO1* mRNA (**Figure 4G**) and protein (**Figure S1F**) expression. We also found that Ectopic overexpression of HBO1 augmented cell proliferation (EdU incorporation, **Figure 4H**) and migration (**Figure 4I**) in the OS cells.

### Zinc finger protein 384 (ZNF 384) is a possible HBO1 transcription factor

**Figure 5:**
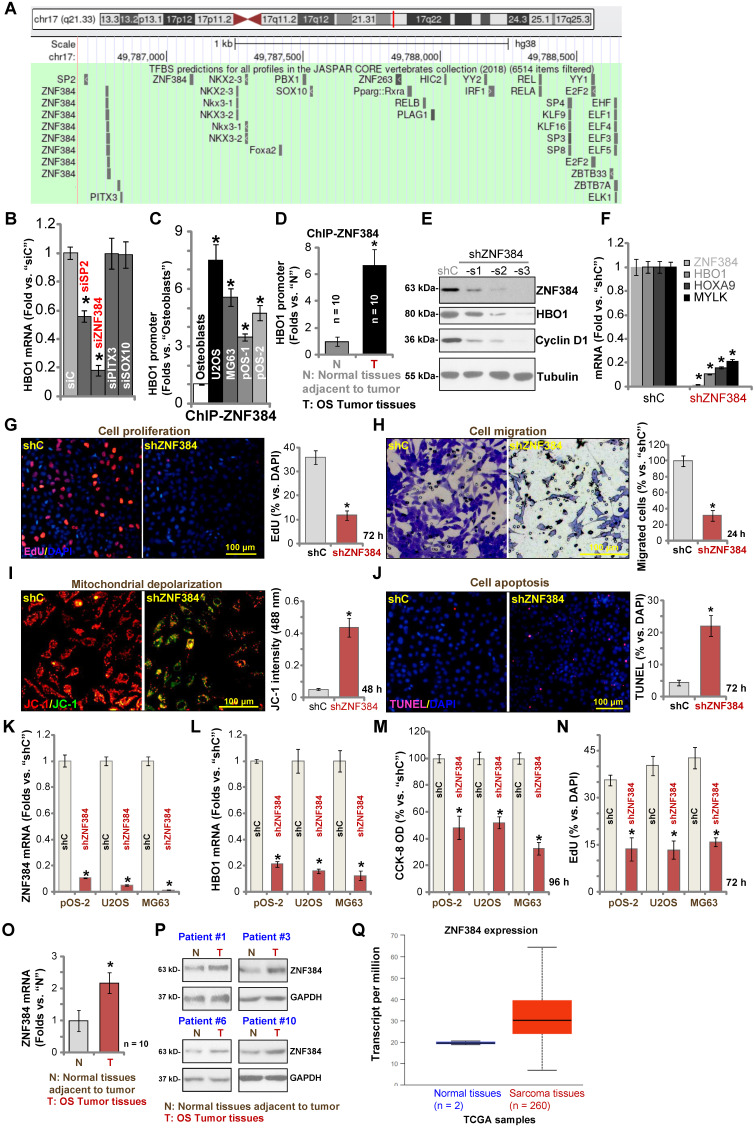
** ZNF384 is a transcription factor of HBO1 in OS cells.** JASPAR database predicted the potential transcription factors of HBO1 (**A**). pOS-1 cells were individually transfected with 200 nM of applied siRNAs or scramble control siRNA (“siC”) for 48h, and *HBO1* mRNA expression was tested by qPCR (**B**). ChIP assay results demonstrated the direct binding between ZNF384 protein and HBO1 promoter in listed human cells and tissues, with results quantified (**C** and **D**). Stable OS cells, including pOS-1 cells, pOS-2 primary cells, as well as the established OS cell lines (U2OS and MG63) that expressed listed ZNF384 shRNAs (“shZNF384”) or the scramble control shRNA (“shC”), were established and expression of listed genes was shown (**E**, **F**, **K**, and **L**); Cells were cultured for applied time periods; cell proliferation (EdU staining assay, **G** and **N**), viability (CCK-8 OD, **M**), migration (**H**), mitochondrial depolarization (by recording JC-1 green monomers intensity, **I**), and apoptosis (nuclear TUNEL staining assay, **J**) were tested. *ZNF384* mRNA and protein expressions in OS tumor tissues (“T”) and in normal tissues adjacent to tumor (“N”) were shown (**O** and **P**). TCGA database showed relative ZNF384 transcript expression in human sarcoma tissues and normal tissues (**Q**). The data were presented as mean ± standard deviation (SD, n = 5). *P < 0.05 vs. “siC”/“shC” cells (**B**, **E**, **G**-**N**).*P < 0.05 vs. “Osteoblasts” (**C**). *P < 0.05 vs. “N” tissues (**D**, **O** and **P**). The experiments were repeated five times with similar results obtained. Scale bar = 100 µm (**G**-**J**).

To study the underlying mechanism of HBO1 upregulation in human OS, we focused on possible transcription factors of *HBO1* by searching in the JASPAR database 54. Through bioinformatical studies, we identified four possible transcription factors with the highest binding affinity to HBO1's promoter (sequences listed in **Table-S1**): SP2, ZNF384 (zinc finger protein 384), PITX3, and SOX10 (**Figure 5A**). Next, siRNA strategy was utilized to knockdown each of the predicted transcription factors. In pOS-1 cells, the applied siRNA resulted in dramatic knockdown of targeted transcription factor (over 80% mRNA knockdown efficiency). Importantly, only SP2 siRNA and ZNF384 siRNA resulted in significant downregulation of *HBO1* mRNA in pOS-1 cells (**Figure 5B**), while the other two failed to do so (**Figure 5B**). Also, ZNF384 siRNA-induced *HBO1* mRNA reduction was significantly more potent than that of SP2 siRNA (**Figure 5B**). These results suggested that ZNF384 could be an important transcription factor of HBO1 in OS cells.

Chromatin immunoprecipitation (ChIP) assay results in **Figure 5C** confirmed that ZNF384 directly binds HBO1 promoter in human osteoblasts and in established (U2OS and MG63 lines) and primary OS cells (pOS-1 and pOS-2). More importantly, there was a significant increase of ZNF384-HBO1 promoter binding in OS cells (*P* < 0.05 vs. osteoblasts) (**Figure 5C**). Also, ZNF384-HBO1 promoter binding was over six-time higher in OS tumor tissues (**Figure 1**) than that in normal tissues (*P* < 0.05, **Figure 5D**). These results implied that increased binding between HBO1 promoter and its proposed transcription factor ZNF384 could be one of the primary mechanisms of HBO1 upregulation in OS.

To further test our hypothesis, lentiviral constructs, encoding three different ZNF384 shRNAs (shZNF384-s1/shZNF384-s2/shZNF384-s3, targeting non-overlapping sequences), were individually transduced to pOS-1 cells. Stable cells were established via puromycin selection. Each of the applied shRNA led to significant ZNF384 protein downregulation (**Figure 5E**). Consequently, HBO1 protein expression was dramatically reduced (**Figure 5E**). Protein expression of a known ZNF384-dependent gene, Cyclin D1 49, was significantly downregulated in pOS-1 cells with ZNF384 shRNA (**Figure 5E**). Of the applied shRNAs, shZNF384-s3, led to most dramatic ZNF384-HBO1 downregulation, and this shRNA was selected for further studies. As shown, ZNF384 shRNA (shZNF384-s3) induced robust downregulation of *HBO1* mRNA and HBO1-dependent mRNAs, *MYLK* and* HOXA9*, in pOS-1 cells (**Figure 5F**). Functional studies in pOS-1 cells showed that ZNF384 silencing resulted in inhibition of proliferation (decreased EdU-positive nuclei ratio, **Figure 5G**) and migration (**Figure 5H**), mitochondrial depolarization (JC-1 green monomers accumulation, **Figure 5I**), and cell apoptosis (TUNEL ratio increase, **Figure 5J**), which mimicked the actions by HBO1 shRNA/KO. Importantly, ZNF384 shRNA-induced anti-OS cell activity was largely attenuated by ectopic HBO1 expression using the HBO1-expressing construct (OE-HBO1, see **Figure 4**) as shown in **Figure S3A**-**E**. These results implied that HBO1 downregulation should be the major reason of ZNF384 shRNA-induced anti-cancer actions in OS cells.

Similarly, in pOS-2 primary cells and established OS cells (U2OS and MG63 lines), transfection of the lentiviral ZNF384 shRNA construct (shZNF384) resulted in *ZNF384* mRNA (**Figure 5K**) and protein (**Figure S1G**) silencing, causing *HBO1* mRNA reduction (**Figure 5L**). ZNF384 shRNA induced significant viability (CCK-8 OD) reduction (**Figure 5M**) and proliferation inhibition (EdU staining, **Figure 5N**) in the primary and established OS cells.

We also tested ZNF384 expression in OS cells and tissues. *ZNF384* mRNA and protein expressions were elevated in U2OS, MG63, pOS-1 and pOS-2 cells, when compared to its levels in osteoblasts (**Figure S1H**). Furthermore, as shown in **Figure 5O**, *ZNF384* mRNA levels in OS tumor tissues (“T”, see **Figure 1**) were significantly higher than those in normal tissues (“N”, see **Figure 1**) (*P* < 0.05 vs. “N” tissues). ZNF384 protein upregulation was also detected in OS tumor tissues from four representative patients (Patient #1/#3/#6/#10) (**Figure 5P**). Furthermore, TCGA database showed ZNF384 transcript is upregulated in human sarcoma tissues (**Figure 5Q**). Therefore, HBO1 expression is correlated with ZNF384 expression in human OS tissues. ZNF384 upregulation, as well as the increased binding between ZNF384 andHBO1 promoter, could be the primary mechanisms of HBO1 upregulation in human OS.

### HBO1 is required for OS xenograft growth in mice

**Figure 6:**
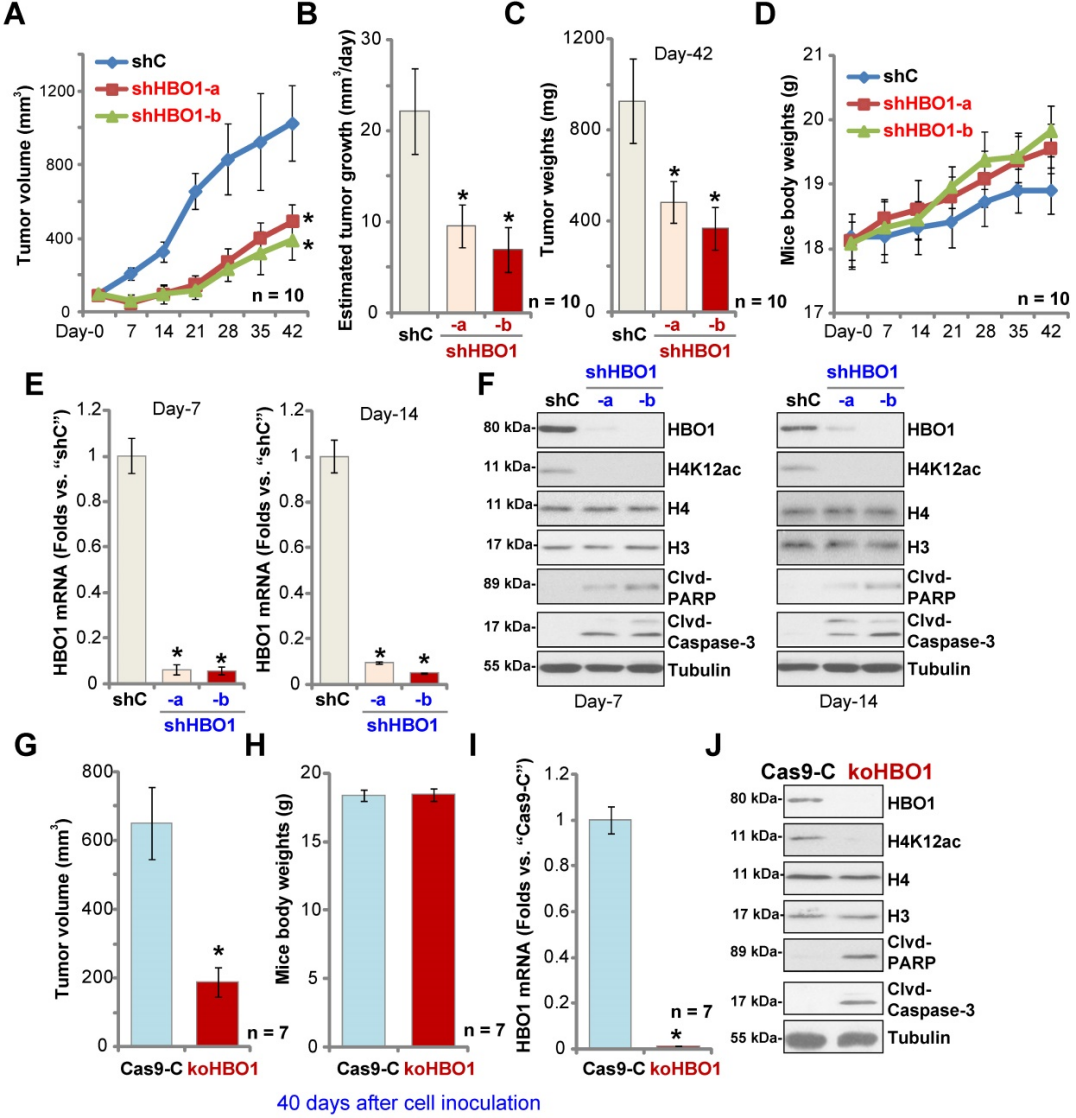
** HBO1 is required for OS xenograft growth in mice.** The pOS-1 xenografts-bearing SCID mice were subjected to intratumoral injection with HBO1 shRNA lentivirus (shHBO1-a/shHBO1-b) or scramble control shRNA lentivirus (“shC”); tumor volumes (**A**) and mice body weights (**D**) were recorded every seven days, and estimated daily tumor growth was calculated by using the described formula (**B**). At experimental Day-42, all tumors were separated through surgery and weighted individually (**C**). Expression of *HBO1* mRNA (**E**) and listed proteins (**F**) in indicated OS xenograft tissues was shown. The stable pOS-1 cells expressing the CRISPR/Cas9-HBO1-KO construct (ko-HBO1 cells) or CRISPR/Cas9 control construct (“Cas9-C”) were *s.c.* injected to the flanks of SCID mice. Forty days after cell inoculation, tumors were separated and measured (**G**); Mice body weights were recorded (**H**); Expressions of *HBO1* mRNA (**I**) and listed proteins (**J**) were shown. Data were presented as mean ± standard deviation (SD). *P < 0.05 vs. “shC”/“Cas9-C” tumors.

To study the potential effect of HBO1 on OS cell growth *in vivo*, a mice xenograft model was utilized. As described, pOS-1 primary cells were *s.c.* injected to the flanks of SCID mice. Xenograft tumors were established within three weeks, with tumor volume close to 100 mm^3^ (“Day-0”). The pOS-1 xenografts-bearing mice were randomly assigned into three groups (10 mice per group) receiving intratumoral injection of shHBO1-a, shHBO1-b (HBO1 shRNA lentivirus) or scramble control shRNA lentivirus (“shC”). Tumor growth curve shown in **Figure 6A** demonstrated that pOS-1 xenografts with shHBO1 injection grew significantly slower than xenografts with shC lentivirus. By calculating estimated daily tumor growth using the formula: (Tumor volume at Day-42 - Tumor volume at Day-0)/42, we found that the growth of HBO1 shRNA-injected pOS-1 xenografts was significantly inhibited (**Figure 6B**). At experimental Day-42, tumors were all separated and individually weighted. Xenografts receiving HBO1 shRNA injection were much lighter than those with control shRNA injection (**Figure 6C**). There was no significant difference in the animal body weights within the three groups (**Figure 6D**). We also failed to detect any apparent toxicities.

To test signaling changes *in vivo*, one tumor of each group was separated at experimental Day-7 and Day-14. A total of six xenografts were obtained and tumor tissues were tested by qPCR and Western blotting. As shown, *HBO1* mRNA levels decreased over 90% in HBO1 shRNA-injected pOS-1 xenografts (**Figure 6E**). HBO1 protein expression and H4 acetylation decreased as well in the pOS-1 xenografts with HBO1 shRNA (**Figure 6F**), at which the cleavages of caspase-3 and PARP were detected (indicating apoptosis activation, **Figure 6F**). Levels of total H3 and H4 histones were unchanged (**Figure 6F**). Therefore, HBO1 shRNA lentivirus injection silenced HBO1 and inhibited pOS-1 xenograft growth in SCID mice.

Next, stable pOS-1 cells expressing the CRISPR/Cas9-HBO1-KO construct (ko-HBO1 cells, see **Figure S2**) and cells with the CRISPR/Cas9 control construct (Cas9-C, see **Figure S2**) were *s.c.* injected to the flanks of SCID mice. Forty days after cells inoculation, xenograft tumors were separated and measured. As shown, the volumes of xenografts of ko-HBO1cells were dramatically lower than xenografts of Cas9-C control cells (**Figure 6G**). Again, no difference was found in mice body weights between the two groups (**Figure 6H**). After analyzing the tumor tissues, we showed that *HBO1* mRNA (**Figure 6I**) and protein (**Figure 6J**) expressions were almost completely depleted in ko-HBO1 xenografts, and we detected H4 acetylation inhibition as well as cleavages of caspase-3 and PARP (**Figure 6J**). H3 and H4 histones were unchanged (**Figure 6J**). These results further confirmed that HBO1 is required for OS xenografts growth in SCID mice.

### The anti-OS activity by a HBO1 inhibitor WM-3835

**Figure 7:**
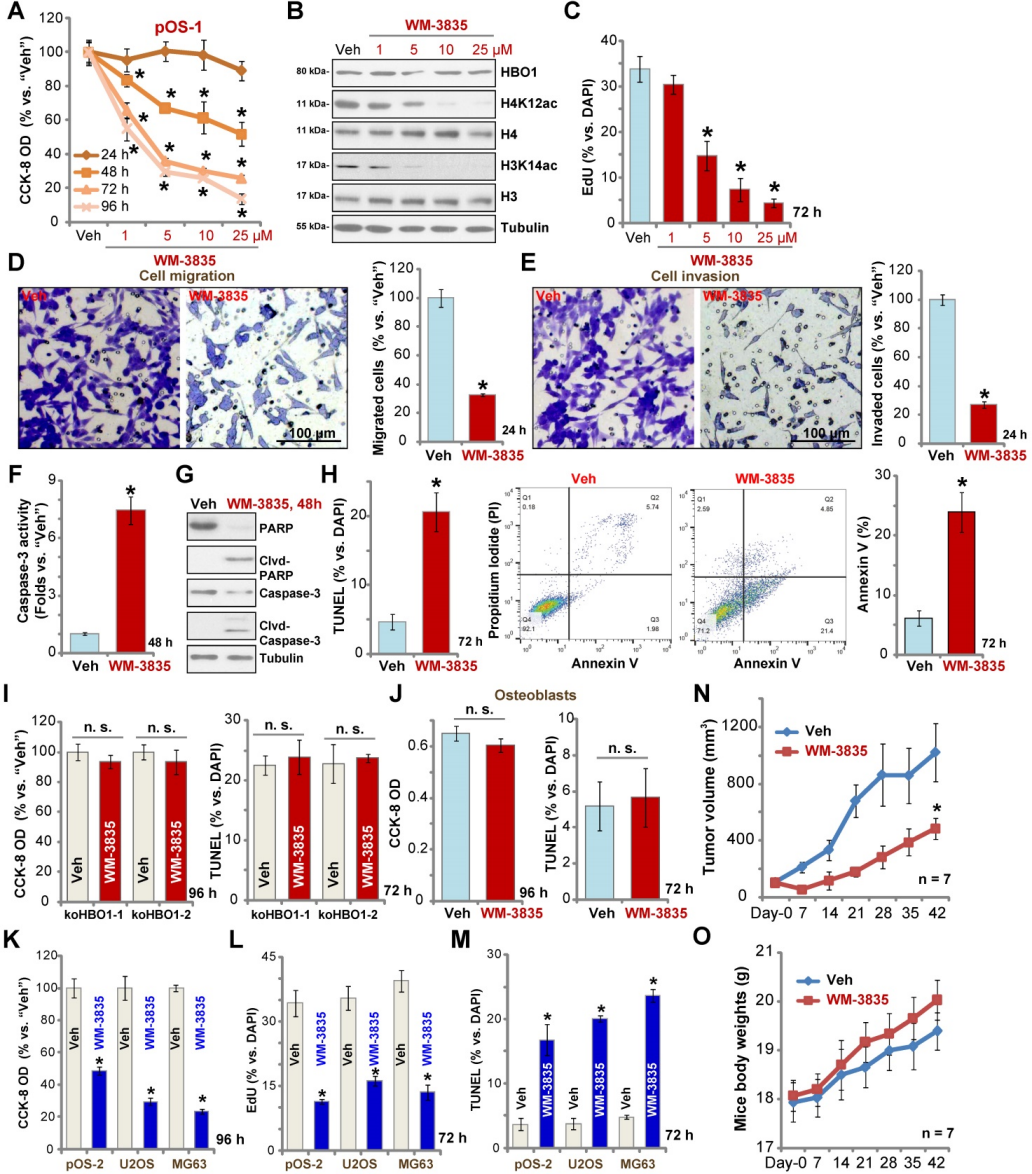
** The anti-OS activity by a HBO1 inhibitor WM-3835.** The pOS-1 cells were treated with WM-3835 (at 5 µM, expect for **A-C**) or vehicle control (“Veh”, 0.5% DMSO), and cells were further cultured in complete medium for indicated time periods. Cell viability (CCK-8 OD, **A**), expression of listed proteins (**B**), cell proliferation (by recording EdU-positive nuclei ratio, **C**), migration (**D**), and invasion (**E**) were tested; Caspase activation (**F** and **G**) and cell apoptosis (nuclear TUNEL staining and Annexin V FACS assays, **H**) were tested as well. The stable HBO1-KO pOS-1 cells, koHBO1-1 and koHBO1-2 (**I**) or the human osteoblasts (“Osteoblasts”, **J**) were treated with WM-3835 (5 µM) or vehicle control for applied time periods, cell viability and apoptosis were tested by CCK-8 and TUNEL staining assays, respectively. The pOS-2 primary cells, as well as the established OS cell lines, U2OS and MG63, were treated with WM-3835 (5 µM) or vehicle control for applied time periods, cell viability, proliferation and apoptosis were tested by CCK-8 (**K**), nuclear EdU staining (**L**) and TUNEL staining (**M**) assays, respectively. The pOS-1 xenografts-bearing SCID mice were subjected to* i.p.* injection of WM-3835 (10 mg/kg, daily for 21 days) or vehicle control (“Veh”); tumor volumes (**N**) and mice body weights (**O**) were recorded every seven days. The data were presented as mean ± standard deviation (SD). *P < 0.05 vs. “Veh” treatment. The *in vitro* experiments were repeated five times with similar results obtained. “n. s.” stands for no statistical difference (**I** and **J**). Scale bar = 100 µm (**D** and **E**).

WM-3835, a small-molecular HBO1 inhibitor, was synthesized based on the structure in an early study 33, and its anti-OS cell activity was tested. CCK-8 viability assay results showed that WM-3835 inhibited pOS-1 cell viability in a concentration-dependent manner (**Figure 7A**). Furthermore, the HBO1 inhibitor required at least 48 h to exert a significant anti-survival activity, displaying a time-dependent manner as well (**Figure 7A**). Western blotting studies demonstrated that WM-3835 did not alter HBO1 protein expression, but suppressed H4K12ac-H3K14ac in a dose-dependent manner (**Figure 7B**). Expressions of total H3 and H4 histones were unchanged (**Figure 7B**). Furthermore, WM-3835 inhibited pOS-1 cell proliferation dose-dependently (EdU-positive nuclei ratio, **Figure 7C**). Titration experiments showed that 5 µM of WM-3835 resulted in robust viability reduction of pOS-1 cells (**Figure 7A**), while H3-H4 de-acetylation (**Figure 7B**) and proliferation inhibition (**Figure 7C**) were observed. Thus this concentration (close to IC-50, **Figure 7A**) was selected for following studies.

We found that WM-3835 (5 µM) downregulated *MYLK-HOXA9* mRNA expression (**Figure S1I**) in pOS-1 cells. It also potently inhibited pOS-1 cell migration (**Figure 7D**) and invasion (**Figure 7E**). Additionally, caspase activation (**Figure 7F**) and cleavages of caspase-3, caspase-9, and PARP (**Figure 7G**) were detected in WM-3835-treated pOS-1 cells. Further confirming cell apoptosis activation by WM-3835, TUNEL-positive nuclei were significantly increased following WM-3835 treatment in pOS-1 cells (**Figure 7H**). Additionally, Annexin V-positive cell number was increased as well (**Figure 7H**). These results confirmed the significant anti-OS activity of HBO1 inhibitor WM-3835.

Importantly, in HBO1-KO pOS-1 cells, koHBO1-1 and koHBO1-2 (see **Figure S2**), treatment with WM-3835 (5 µM) failed to induce apoptosis and reduction of viability (CCK-8 and TUNEL assays, **Figure 7I**). Similarly, in HBO1-low human osteoblasts (see **Figure 1**), treatment with WM-3835 (5 µM) failed to induce significant cytotoxicity and apoptosis (**Figure 7J**). These results suggest that WM-3835 induced anti-OS cell activity through HBO1 inhibition. In pOS-2 primary cells and established OS cell lines (U2OS/MG63), WM-3835 (5 µM) potently inhibited cell viability (CCK-8 OD, **Figure 7K**) and proliferation (EdU ratio reduction, **Figure 7L**), while provoking apoptosis (TUNEL-positive nuclei ratio increase, **Figure 7M**).

The activity of WM-3835 *in vivo* was tested as well. The pOS-1 cells were injected* s.c.* to the flanks of SCID mice to establish OS xenografts. As demonstrated, daily *i.p.* injection of WM-3835 (at 10 mg/kg body weights for21 days) potently inhibited pOS-1 xenograft growth in SCID mice (**Figure 7N**). WM-3835 did not provoke apparent toxicities to the experimental animals, and no significant difference was found in mice body weights between two groups (**Figure 7O**). Therefore, *i.p.* injection of WM-3835 successfully inhibited OS xenografts growth in mice.

## Discussion

Histone acetylation is a covalent modification of lysine residues in histone tails 19. This process is vital for the regulation of gene expression 19. HBO1 (KAT7/MYST2) belongs to the MYST family of acetyltransferases, and is responsible for the majority of H4 acetylation 23. HBO1 is evolutionarily conserved, and functions in the context of a multi-protein HAT complex 23, 55. HAT complex is composed of JADE1/2/3, HBO1, ING4/5, BRPF and Eaf6, and it regulates DNA replication, gene transcription, expression, cell cycle progression, and other important cellular functions 23.

HBO1 plays an essential role in gene transcription, DNA replication, and is hence implicated in cancer initiation and progression 22, 23, 33-35. Quintela* et al.* reported that in ovarian cancer cells, HBO1 acetylated histone H4 through the co-regulator JADE2 to regulate mechano-transduction pathways and cell elasticity 30. In embryonic stem cells, association of HBO1 and Niam (Nuclear Interactor of ARF and Mdm2) is essential in early development, cell survival, as well as cancer progression 56. Recently, Taniue and colleagues reported that TUSC3 expression induced by HBO1 via histone acetylation is critical for colon cancer cell proliferation 57. Bai *et al.* also reported that microRNA-639 silenced HBO1 via blocking Wnt/β-catenin pathway to inhibit human hepatocellular carcinoma cell proliferation and migration 58.

The results of this study suggested that HBO1 could be an important novel oncogene for OS cell progression. HBO1 is associated with poor overall survival as it was overexpressed in OS tissues, whereas low expression was detected in parecancer normal tissues and cultured human osteoblasts. In established and primary human OS cells, HBO1 silencing induced by shRNA potently inhibited cell viability, proliferation, migration and invasion, but promoted significant apoptosis activation. Furthermore, CRISPR/Cas9-induced HBO1 KO exerted robust anti-OS cell activity. Conversely, ectopic overexpression of HBO1 augmented OS cell proliferation and migration. *In vivo*, HBO1 silencing by intratumoral injection of HBO1 shRNA lentivirus potently inhibited OS xenograft growth in SCID mice. Furthermore, growth of HBO1-KO pOS-1 xenografts was largely inhibited in SCID mice. Therefore, HBO1 presented vital oncogenic activities required for OS cell progression *in vitro* and *in vivo*, and it should be considered as a novel therapeutic target for OS.

ZNF384, a C2H2-type zinc finger protein, is a transcription factor that regulates the transcription of extracellular matrix genes 59. Studies have proposed that ZNF384 could be a potential oncogene and it is overexpressed in various human cancers 49, 60-62. Zhong *et al.* showed that ZNF384 is important for acute leukemia (ALL) progression and it fused with the TET family genes including Ewing sarcoma breakpoint region 1 (EWSR1), TATA box binding protein-associated factor (TAF15), and transcription factor 3 (TCF3) 62. ZNF384 overexpression promoted melanoma cell migration 61, while in cervical cancer, ZNF384 bound to APOBEC3B's promoter 60. He *et al.* found that ZNF384 is overexpressed in hepatocellular carcinoma (HCC) and it promoted HCC cell growth by upregulating cyclin D1 expression 49.

In the present study, our results proposed that ZNF384 could be an important transcription factor that directly binds to HBO1 promoter. In OS, increased binding of ZNF384-HBO1 promoter could be the primary mechanism of HBO1 upregulation. In support of our conclusion, we found that shRNA-induced silencing of ZNF384 downregulated HBO1 expression and the target mRNAs in OS cells. Moreover, ZNF384 shRNA produced significant anti-OS cell activity, which mimics the activity induced by HBO1 silencing/KO. Importantly, ectopic overexpression of HBO1 significantly attenuated ZNF384 shRNA-induced anti-OS cell activity.

WM-3835 is a cell permeable small molecule HBO1 inhibitor 33. WM-3835 binds directly to the acetyl-CoA binding site of HBO1 33. It is a highly specific inhibitor of HBO1, as it makes additional interactions with HBO1 protein surface. Specifically, WM-3835 phenol forms a hydrogen-bonding network with HBO1's Glu525 and Lys488. The two amino acids are specific for HBO1, but not conserved throughout the MYST family proteins. Therefore, WM-3835 can exert robust and selective HBO1 inhibition 33.

We found that WM-3835 activated apoptosis while inhibited OS cell proliferation, migration and invasion. Notably, the activity of WM-3835 was nullified in HBO1-KO OS cells. Also, the HBO1 inhibitor was non-cytotoxic in HBO1-low human osteoblasts, which indicates that the anti-OS cell efficacy of WM-3835 is primarily through HBO1 inhibition. Importantly, *i.p.* injection of a single dose of WM-3835 potently inhibited pOS-1 xenograft growth in SCID mice, highlighting the potential translational value for this compound. Therefore, targeting HBO1 by WM-3835 could be a novel therapeutic strategy against human OS.

Despite significant progresses that have been made in surgical techniques and new adjuvant chemotherapies, over 30% of OS patients still die from lung metastases within five years of diagnosis 3, 8, and the prognosis of advanced and recurrent OS patients remains extremely poor 3, 8. The results of this study indicated that targeting HBO1 via pharmacological (WM-3835 or others) or genetic methods could be a valuable novel therapeutic approach against human OS. Moreover, we here identified ZNF384 as a potential transcription factor of HBO1. Increased binding between ZNF384 and HBO1 promoter could be a key mechanism of HBO1 upregulation in OS. Considering that HBO1 is upregulated in several other human cancers (gastric cancer, bladder cancer, etc.) 22, 23, 33 and functions as a potential oncogene 22, 23, 33, it certainly will be interesting to further test the potential anti-cancer activity of HBO1 genetic silencing or pharmacological inhibition in other cancer models.

## Supplementary Material

Supplementary information, figures and table.
