# Supplementary material for: The histone acetyltransferase HBO1 functions as a novel oncogenic gene in osteosarcoma
Source: Theranostics. 2021 Mar 4;11(10):4599–615. doi: 10.7150/thno.55655 (PMC7978299; doi:10.7150/thno.55655)
Supplement: Supplementary file 1 — Supplementary information, figures and table. [file thnov11p4599s1.pdf]

Table S1: sequences utilized in this study.

**qPCR primers**	Forward5’-3’	Reverse 5’-3’
Hoxa9	AGAATGAGAGCGGCGGAGACAA	CTCTTTCTCCAGTTCCAGGGTC
HBO1	TTTTGGCCGCTATGAACTG	GGAGGATTGTCTGGCTCTTC
GAPDH	GTCTCCTCTGACTTCAACAGCG	ACCACCCTGTTGCTGTAGCCAA
MYLK	GAGGTGCTTCAGAATGAGGACG	GCATCAGTGACACCTGGCAACT
**shRNA**		
shHBO1-a	CCGGGCCTTCTTCTGAGTCTGACATCTCGAGATGTCAGACTCAGAAGAAGGCTTTTT	
shHBO1-b	CCGGCTCGTTCATCTGGTTCAGAAACTCGAGTTTCTGAACCAGATGAACGAGTTTTT	
shZNF384-s1	CCGGGCCTTCACACAACTCTCCAATCTCGAGATTGGAGAGTTGTGTGAAGGCTTTTTG	
shZNF384-s2	CCGGGATCGAGAACACAATGTTCATCTCGAGATGAACATTGTGTTCTCGATCTTTTT	
shZNF384-s3	CCGGCGGCAACACAACAAAGATAAACTCGAGTTTATCTTTGTTGTGTTGCCGTTTTTG	
**siRNA**		
SOX10	CCGUAUGCAGCACAAGAAA	
SP2	UGCAGACCAUCAACAUCAA	
ZNF 384	AAUUACUUUAUAACAACUGGU	
PITX3	UUUAUUUCAUUUAUCUUUGAA	

HBO1 promoter region

GCAGGAGAATCGCCTGAACCCGGGAGGCGGAGGCTGCGGTGAGCCGAGCTCGTGCCATTGC
CCTCCAGCCTGGCAACAAGAGCAAAACCCTGTCTCAGAAAAAAAAAAAAAAAAAAGAAAGGC
TGGTATGGCTGGGAGTGGTGGCTCACACCTTAATCCCAGCACTCTGGAAGGCCGAGACAGTA
GTTCAGCTCAGTAGGTCGAGACCAGCCTGGGCAGCATGGTGAAACTCTGTCTCTACAAAAAAT
ACAAAAATTAGCCAGGTGTGGTGGCACACGCCTGCAGTCCCAGCTACTCGGGAGGCTGAGGC
AAGAGAATCCCTTGAGCCCACGAGGTGGAGCTTGCAGTGAACTGACATCCAGCCATTGCACT
CCAGCCTGGGCAACACAGCAAGACTCTGTCTTGAAAGAAAAAAAAAGAAGAGAGAGAAAAA
AAGTAGGATGGTATGAAATAACTGAGATATGCTCTTAATGTAAGAGATCCTTACCACCAGTTGG
GGAGCCAGAACATTCAAATGTAACCTCAGGAACATATCCGTGAGTGCAAAATAACAGAGTAAT
GTATGAAATTAAATGCTTAAGTGGTGAGGGGCTACATGAAATTAAATGCTTAAGTGGTGAGAA
GTAATGTGAACCATTTTCAAATAAGTTCTTACGTGCAGTTCAACCTAAGTAGGTTATCAAAGGT
ATTTATACTAAGCACTAAAAGCTAAGTATTAAATACTAAAAGCTGCCTGTGTCAACACTGGCCC
AGGACATCCCCAAGGAGAGTGCTGGCTAAAGGTCTGGAACTTTAAATCTTTAGAGTTCTTTAC
TTAGCACATCAATCAGATATTTACCTTCTGCTTCTTTGTTCTTTAGCCCTGCTCCTGGGGGAAAT
CTCCATCTTAACGGGGCCTCAGTTTGAAATTGGGGTTCGGGATCCTAACTTAATGACTGAGCG
TGCCTGGGTTCCACCCAGTCGCAATTGGTCAAGACTTGGTCCTCTCAAGGCCTTTCCTCCGGA
GAGGTTAAATCCTCAACGCCTGCTCTCCACTTTCCAGCCTTTTCTTAAGGCTCTTCTTTCAGCCT
AGTATCTGTGACCAACTTCTCCTTCCTAGGCCCTCTCATTATTATTTAAATGTCCATTCTTTTGGG
GGCTTCAACTTCACTAAAAACATTCCTTCCTCTTCCTCCGCCTCGTAACTCTCTGCTCAAGCATC
TGGATGCCAGAGGTCATTTGAGCTAAATGGGGTTCAAAGCTTTTCTGACTCTGGTACTGCATTC
CCCACTTTCAAATCACATTTTTCACCCCAGGGCCCCCAGTGGTCTCAAACCCAAACACAGTGC
GGTTAGGAAGTGGGCACTCGCCACAGAGCTAAATTGGGAATTATTTGACCTGCAGCTTCTGGA
GAATGGTTTTGCCTCCTCCCCGCGACATCAACTAACTCACTTGCTGTTAGCTAAGGGCAAGGA
CAATGTCTCCTCTACCAGACAGGAAACTTGATGGCGGACGCTATCCGTTAGTTCCTTTTTCAAT
TCCCCATTCCCACCCCTTCGAGACCACGATGTTTCTTAAAATACAAAGTTTCTTTTACAAAACAT
CCCTCCGCAACTTTGAGTTTATATAATAAACGGAAACGTCCTTTAAACAATCAGGCTACGAAG
GGGTTAAATCTAATCTCAGCAACCTGATGGATTCTGGGGGGTTTCCTTCCTCGACTCTAGAAAT
TAAAACGAAATTTTGCCCAAGGTAAAGATGAGAACCGTTTACATCAACTGTCAGGCTTCTGCC
CCAAACAAGAATGGCTACCCACGCCCACAAACAGAGCCACTCACGTAACTAAAAATGGTTGG
CTCCGGGTTCCACCACTGGAGTCACTTTCTGCCCAACTTCAAAATGGCGCCCACTAGCTTCACC
AAAAGGATCGAACTTCCCAGCACCCTCTCTGGCCCGTAACGTCAGGTGACGCGAGACCCAGC
CGGAAGTGAAGGAAAAAGCGCTTCAGCCCGCGGCGCCTGCGCAGAACGCTCCAGACGCTGA
GAGGCAGGAGGCACTAGGGATCGTCCGCAGGATTGGGACTGATACAGAGGCCGCCACGGAG
CCCGCCGGAGCCACCGTT

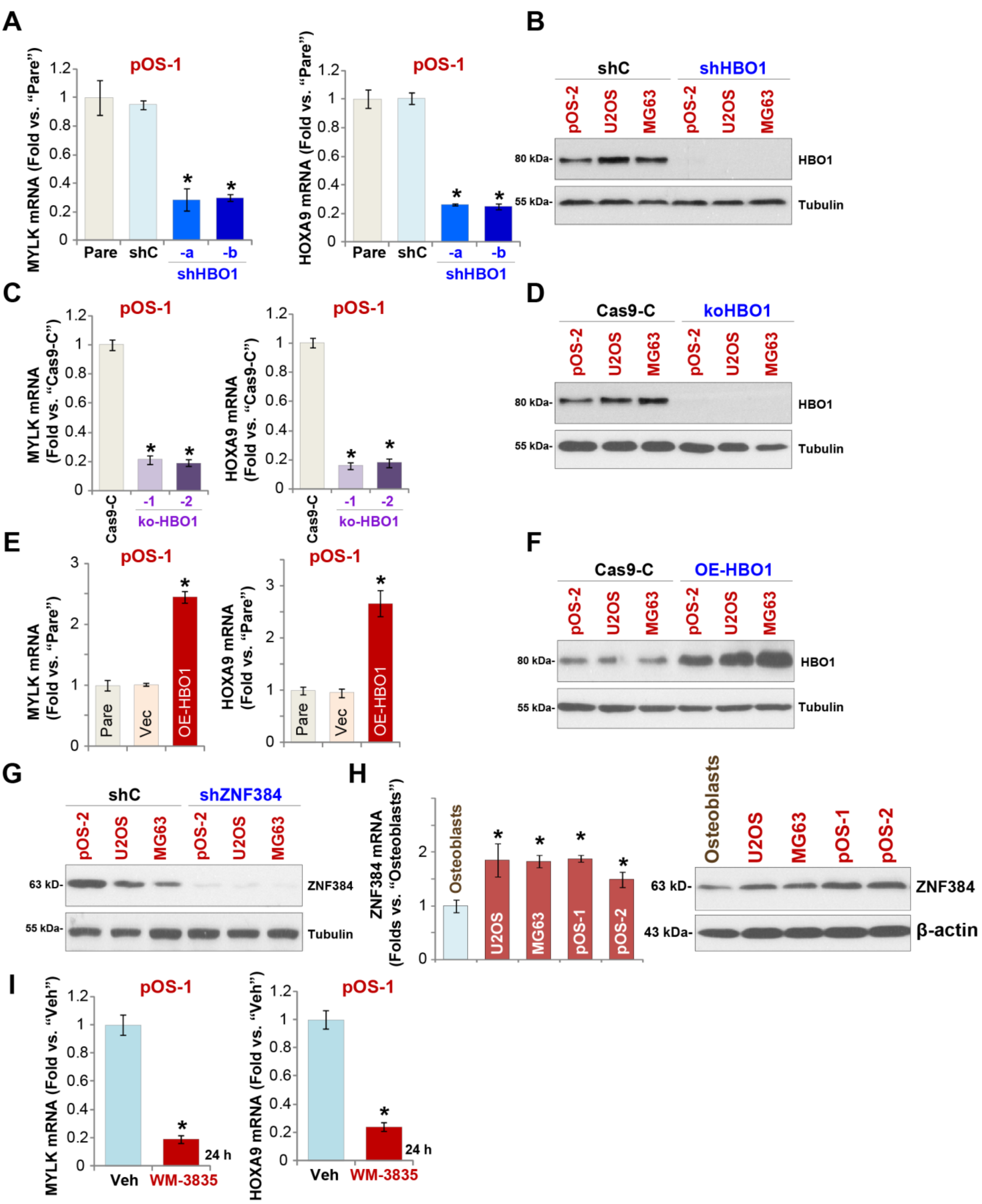

Figure S1. Expressions of *MYLK-HOXA9* mRNA and listed proteins in primary OS cells (pOS-1 and pOS-2) and established OS cell lines (U2OS and MG63), with described genetic modifications, were tested by qPCR and Western blotting analyses (**A**-**G**). *ZNF384* mRNA and protein expressions in established OS cell lines (U2OS and MG63), primary human OS cells

(pOS-1 and pOS-2), as well as in primary human osteoblasts ("Osteoblasts") were shown (**H**). The pOS-1 cells were treated with WM-3835 (5 μM) or vehicle control for 24h, *MYLK-HOXA9* mRNA expression was tested (**I**). The data were presented as mean ± standard deviation (SD, n=5). * ***P***< 0.05 vs. "Pare"/"Cas9-C" cells, "Osteoblasts", or "Veh" treatment. The experiments were repeated five times with similar results obtained.

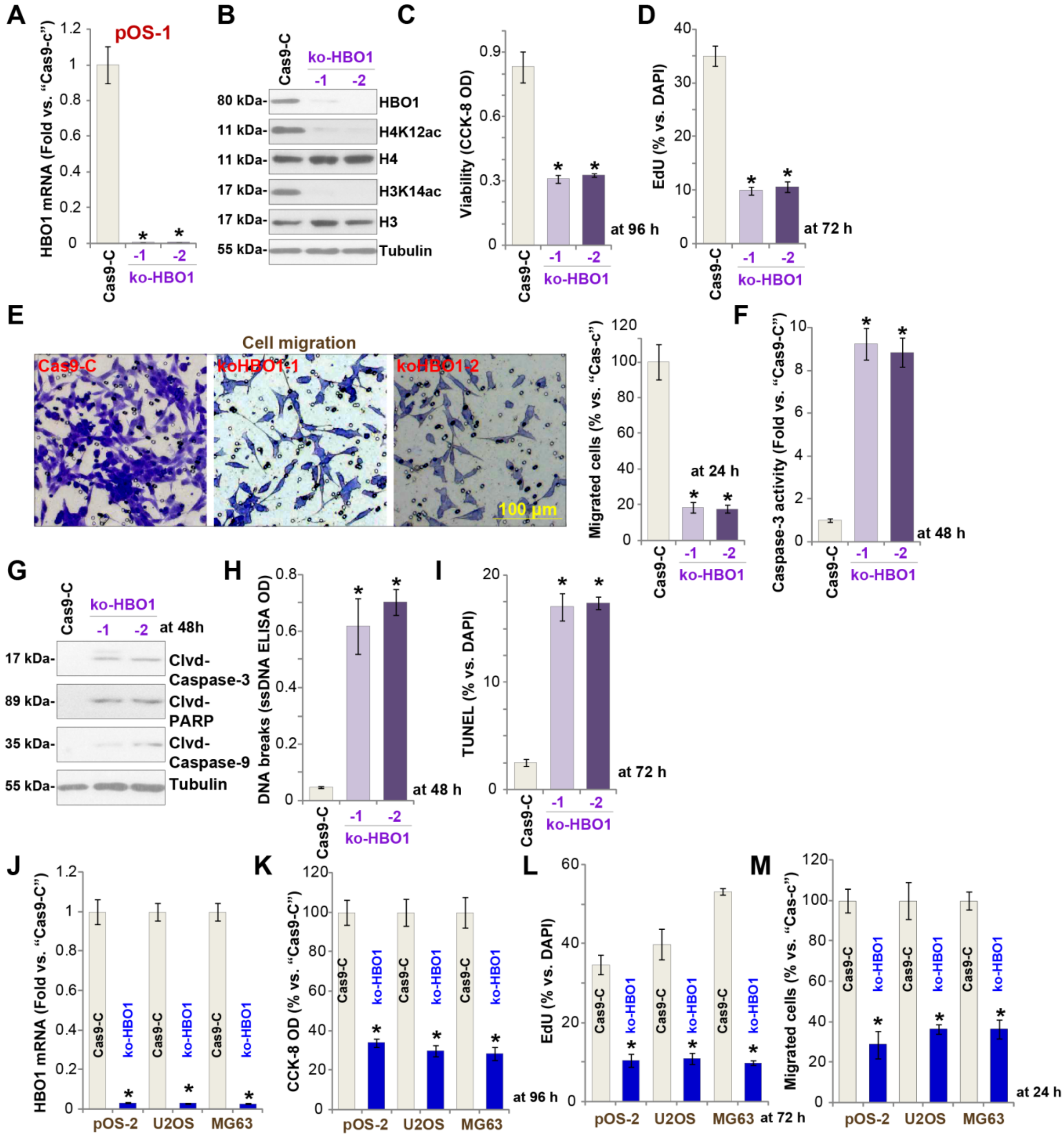

Figure S2. Stable primary OS cells (pOS-1 and pOS-2) and established OS cell lines (U2OS and MG63) with the lentiviral CRISPR/Cas9-HBO1-KO construct "koHBO1", or the CRISPR/Cas9 control construct "Cas9-C", were established. Expression of *HBO1 mRNA* and listed proteins was shown (**A**, **B** and **J**); Cells were cultured for applied time periods; cell viability (**C** and **K**), proliferation (by recording EdU-positive nuclei ratio, **D** and **L**), and migration (**E** and **M**) were tested by the assays mentioned, with data quantified. The caspase-PARP activation (**F** and **G**), single strand DNA (ssDNA) contents (**H**), and cell

apoptosis (by recording nuclear TUNEL ratio, **I**) were tested as well. The data were presented as mean ± standard deviation (SD, n=5). * $P < 0.05$ vs. "Cas9-C" cells. The experiments were repeated five times with similar results obtained. Scale bar= 100 μm (**E**).

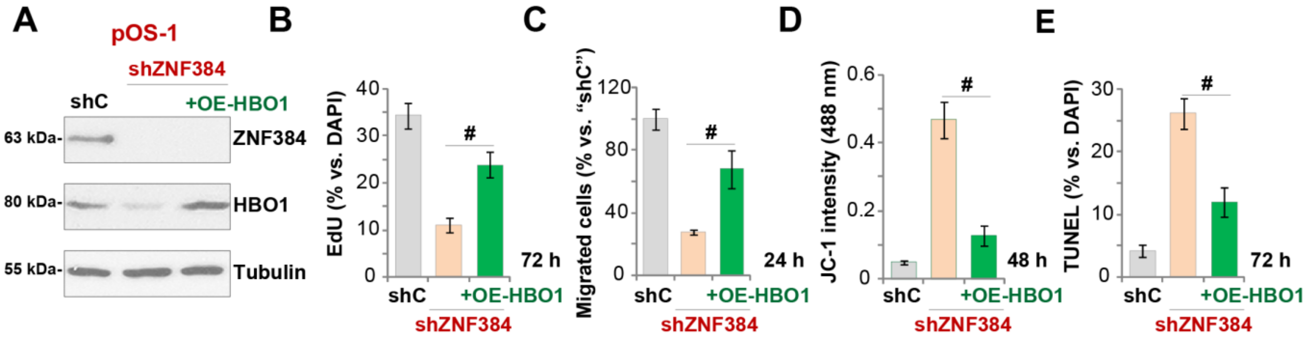

Figure S3. Stable pOS-1 cells expressing ZNF384 shRNA ("shZNF384") were further transduced with or without the lentiviral construct encoding full-length *HBO1 cDNA* ("OE-HBO1"), and control cells were with the scramble control shRNA ("shC"). Expressions of listed proteins were shown (**A**); Cells were cultured for applied time periods; cell proliferation (EdU staining assay, **B**), migration (**C**), mitochondrial depolarization (by recording JC-1 green monomers intensity, **D**), and apoptosis (nuclear TUNEL staining assay, **E**) were tested. $^{\#}$***P***< 0.05. The experiments were repeated five times with similar results obtained.
